# Obesity in women living with HIV aged 45–60 in England: An analysis of the PRIME study

**DOI:** 10.1111/hiv.13242

**Published:** 2022-02-18

**Authors:** Asma N. Ashraf, Hajra Okhai, Caroline A. Sabin, Lorraine Sherr, Katharina Haag, Rageshri Dhairyawan, Richard Gilson, Fiona Burns, Fiona Pettitt, Shema Tariq

**Affiliations:** ^1^ Institute for Global Health University College London London UK; ^2^ Mortimer Market Centre Central North West London NHS Foundation Trust London UK; ^3^ Health Protection Research Unit in Blood Borne and Sexually Transmitted Infections at University College London London UK; ^4^ Barts Health NHS Trust London UK; ^5^ Blizard Institute Queen Mary University of London London UK; ^6^ Royal Free London NHS Foundation Trust London UK; ^7^ Positively UK, London London UK

**Keywords:** HIV, menopause, obesity, women

## Abstract

**Objectives:**

Menopause contributes to weight gain in women. We explored factors associated with obesity in women with HIV aged 45–60 years.

**Methods:**

The present study is an analysis of cross‐sectional questionnaire and clinic data from the Positive Transitions Through the Menopause (PRIME) Study. We categorized body mass index (BMI) as normal/underweight (< 25 kg/m^2^), overweight (25–29.9 kg/m^2^) and obese (> 30 kg/m^2^). We used logistic regression to explore demographic, social, lifestyle and clinical factors associated with BMI.

**Results:**

We included 396 women in this analysis. Median age was 49 years [interquartile range (IQR): 47–52]. Most (83.6%) were not UK‐born; the majority (69.4%) were black African (BA). Median (IQR) BMI was 28.6 (24.6–32.6) kg/m^2^; and 110 (27.8%), 127 (32.1%) and 159 (40.1%) of the women were normal/underweight, overweight and obese, respectively. Median (IQR) BMI did not differ in pre‐, peri‐ and post‐menopausal women (*p* = 0.90). In univariable analysis, being non‐UK‐born was associated with BMI > 30 kg/m^2^ [odds ratio (OR) = 1.94, 95% confidence interval (CI): 1.07–3.53]. Compared with BA women, women of other black ethnicities were more likely to be obese (OR = 2.37, 95% CI: 1.02–5.50) whereas white British women were less likely to be obese (OR = 0.34, 95% CI: 0.17–0.68). Current smoking and increasing number of comorbid conditions were associated with increased BMI. We found no association between obesity and socioeconomic status. On multivariable analysis, only ethnicity remained associated with obesity (compared with BA: white British, OR = 0.34, 95% CI: 0.17–0.68; other black, OR = 2.50, 95% CI: 1.07–5.82).

**Conclusions:**

Nearly two‐fifths of women had BMI > 30 kg/m^2^. Obesity was associated with black ethnicities but not with menopausal status. The combination of obesity and HIV may place women at increased risk of co‐morbidities, requiring tailored and culturally appropriate interventions.

## INTRODUCTION

Obesity affects over 650 million people globally, tripling in prevalence since the mid‐1970s. In 2016, 13% of the world's adult population were obese (11% of men and 15% of women) [1].

Weight gain is also an increasingly important issue for people living with HIV; 24.6% of people living with HIV aged ≥ 50 years were found to be obese in a recent UK study [2]. Furthermore, the proportion of people commencing antiretroviral therapy (ART) who are obese increased from 11% in 1998 to 17% in 2010 in a North American cohort [3]. The aetiology of weight gain in HIV is multifactorial with contributory factors including some antiretroviral agents (including integrase inhibitors, especially in combination with tenofovir alafenamide), immune restoration after starting ART, behavioural factors and social inequalities [4, 5, 6, 7].

Weight gain has a significant impact on the health and well‐being of people living with HIV, and is associated with cardiovascular disease (CVD), liver and renal disease [2, 8, 9]. An analysis of pooled data from three randomized controlled trials in the United States demonstrated that women gained more weight on initiation of ART than their male counterparts [8]. It is well recognized that the prevalence of obesity peaks in middle age for both men and women [10], and that menopause, with its attendant hormonal changes, contributes to weight gain and the increase and redistribution of fat mass, although the underlying mechanisms remain unclear [11].

Women living with HIV, similarly to men, are living longer with HIV and are therefore experiencing age‐related events such as menopause [12]. In the UK there are approximately 11 000 women of menopausal age (aged 45–56 years) living with HIV; a five‐fold increase over a 10‐year period [13]. However, data on the prevalence of obesity, and its association with menopause transition, remain scarce among women living with HIV.

In this analysis we use data from the Positive Transitions Through the Menopause (PRIME) Study, a cross‐sectional, mixed‐methods observational study of menopause in women living with HIV in England, to describe the prevalence of obesity, and factors associated with this, in women living with HIV aged 45–60 years.

## METHODS

The PRIME study methods are described in detail elsewhere [14]. In brief, women (defined by sex assigned at birth) aged 45–60 years were recruited from 21 National Health Service HIV clinics across England between February 2016 and June 2017. Women were ineligible if they had experienced surgical menopause, had received chemotherapy or radiotherapy in the last 6 months, or if their last menstrual period was more than 60 months prior to study enrolment. The PRIME Study had ethical approval from the South East Coast‐Surrey Research Ethics Committee (ref. 15/0735).

Self‐completed paper questionnaires collected information on the following: demographic/social factors (age, ethnicity, UK‐born, immigration, employment, relationship and financial status, educational attainment); comorbidities (hepatitis B/C, hypertension, diabetes, CVD, stroke, osteoporosis, breast cancer); current lifestyle [smoking, recreational drug use, alcohol use (assessed using the Alcohol Use Disorders Identification Test, AUDIT‐C, with a score of ≥ 5 considered to be risky)]; psychological distress (Patient Health Questionnaire‐4 score ≥ 3); HIV history (years since HIV diagnosis, most recent CD4 count and HIV viral load); and menopause‐related symptoms and management.

For women who consented, questionnaire data were supplemented with routinely collected data from clinical records, including nadir CD4 count, baseline HIV viral load, height and weight. Menopausal status was determined from self‐reported menstrual pattern (without biological confirmation) and categorized as pre‐menopausal (regular menstruation), peri‐menopausal (irregular periods over the previous 2 years) and post‐menopausal (amenorrhea for 12 months or more). The total number of medical conditions reported by women was used to explore the impact of multi‐morbidity.

Body mass index (BMI) was calculated from height and weight, where available. Women were divided into three categories based on their BMI: normal/underweight (< 25.0 kg/m^2^), overweight (25.0–29.9 kg/m^2^) and obese (≥ 30.0 kg/m^2^).

Demographic, social and HIV‐related characteristics at the time of questionnaire completion were compared across the three BMI groups using χ^2^ and Kruskal–Wallis tests, as appropriate. Factors associated with obesity (BMI ≥ 30.0 kg/m^2^) were assessed using logistic regression. All characteristics associated (*p* < 0.10) with obesity in univariable analyses were included in a multivariable model.

## RESULTS

In total, 592/869 (68.1%) women from the PRIME study consented that the study could access their clinical records. Of these, 396 (66.9%) had height and weight measures available to calculate BMI and were therefore included in this analysis.

Women included (Table 1; *n* = 396) had a median age of 49 [interquartile range (IQR): 47–52]; 21.4%, 47.7% and 30.9% were determined to be pre‐, peri‐ and post‐menopausal, respectively. Over three‐quarters (76.0%) were of black African or other black ethnicity (including mixed black and black Caribbean), followed by 13.7% of white British ethnicity and 7.1% from other ethnic backgrounds. The majority of women were not born in the UK (83.6%) but did have secure immigration status (94.7%). Approximately 1 in 10 women had not completed any education (11.4%), and 38.0% reported not having enough money to cover their basic needs most or all of the time. Over 40% of women (*n* = 155) scored above the cut‐off for psychological distress. Although 15.7% of participants reported risky alcohol use, only 8.3% and 2.3% of women reported current smoking or recent recreational drug use, respectively. Based on clinical data, all women were prescribed ART. The majority of women were on a nonnucleoside reverse transcriptase inhibitor regimen (47.9%), followed by a protease inhibitor regimen (29.3%). Only 13% of women were on an integrase inhibitor‐containing regimen. Nearly 90% (*n* = 353) had an undetectable HIV viral load when last tested, and just over 70% had a T‐cell CD4 count > 500 cells/µL (*n* = 284).

**TABLE 1:** Characteristics of women included in this analysis (with BMI data available)

	All	Obese (BMI ≥ 30.0 kg/m^2^)		
		No	Yes	
*N*	396	237	159	*p*‐value
Age (years) [median (IQR)]	49 (47–52)	49 (47–52)	49 (47–52)	0.20
Ethnicity [*n* (%)]				
Black African	275 (72.4%)	153 (68.0%)	122 (78.7%)	< 0.001
White British	52 (13.7%)	41 (18.2%)	11 (7.1%)	
Other black	26 (6.8%)	9 (4.0%)	17 (11.0%)	
Other	27 (7.1%)	22 (9.8%)	5 (3.2%)	
Menopausal status [*n* (%)]				
Pre‐	83 (21.4%)	53 (22.6%)	30 (19.5%)	0.76
Peri‐	185 (47.7%)	110 (47.0%)	75 (48.7%)	
Post‐	120 (30.9%)	71 (30.3%)	49 (31.8%)	
Born UK [*n* (%)]				
Yes	62 (15.8%)	45 (19.1%)	17 (10.8%)	0.03
No	331 (84.2%)	191 (80.9%)	140 (89.2%)	
Employment status [*n* (%)]				
Full time	200 (51.9%)	109 (47.8%)	91 (58.0%)	0.13
Part time	60 (15.6%)	40 (17.5%)	20 (12.7%)	
None	125 (32.5%)	79 (34.6%)	46 (29.3%)	
Relationship status [*n* (%)]				
No	170 (46.4%)	102 (46.4%)	68 (46.6%)	0.77
Non cohabiting	79 (21.6%)	50 (22.7%)	29 (19.9%)	
Cohabiting	117 (32.0%)	68 (30.9%)	49 (33.6%)	
Completed education [*n* (%)]				
No	45 (12.0%)	24 (10.6%)	21 (14.2%)	0.41
High school	175 (46.7%)	104 (45.8%)	71 (48.0%)	
University	155 (41.3%)	99 (43.6%)	56 (37.8%)	
Money to cover your basic needs [*n* (%)]				
All/most the time	243 (62.0%)	150 (63.8%)	93 (59.2%)	0.53
Some/none of the time	149 (38.0%)	85 (36.2%)	64 (40.8%)	
Smoking status^a^ [*n* (%)]				
No	352 (91.4%)	203 (88.6%)	149 (95.5%)	0.02
Yes	33 (8.6%)	26 (11.4%)	7 (4.5%)	
Recreational drug use^b^ [*n* (%)]				
No	375 (97.7%)	222 (96.5%)	153 (99.4%)	0.08
Yes	9 (2.3%)	8 (3.5%)	1 (0.6%)	
Risky alcohol use^c^ [*n* (%)]				
No alcohol	156 (39.4%)	92 (38.8%)	64 (40.3%)	0.86
No risky alcohol use	178 (44.9%)	106 (44.7%)	72 (45.3%)	
Risky alcohol use	62 (15.7%)	39 (16.5%)	23 (14.5%)	
Psychological distress^d^ [*n* (%)]				
No	195 (56.0%)	125 (58.1%)	72(52.6%)	0.30
Yes	155 (44.0%)	90 (41.9%)	65 (47.4%)	
Number of medical conditions [median (IQR)]	0.0 (0.0–1.0)	0.0 (0.0–1.0)	0.0 (0.0–1.0)	0.10
Last HIV viral load (copies/mL)				
Undetectable	353 (89.1%)	216 (91.1%)	137 (86.2%)	0.12
Detectable	43 (10.9%)	21 (8.9%)	22 (13.8%)	
Last T‐cell CD4 count (cells/µL )				
> 500	284 (71.7%)	168 (70.9%)	116 (73.0%)	0.54
200–500	96 (24.2%)	61 (25.7%)	35 (22.0%)	
< 200	16 (4.0%)	8 (3.4%)	8 (5.0%)	

Note

Missing data: ethnicity, 16; menopausal status, 8; UK‐born, 3; employment status, 11; relationship status, 30; completed education, 21; money to cover basic needs, 4; smoking status, 11; recreational drug use, 12; psychological distress, 44; on combination antiretroviral therapy, 14.

Abbreviations: BMI, body mass index; IQR, interquartile range.

^a^ Current smoking.

^b^ In past 3 months.

^c^ Alcohol Use Disorders Identification Test (AUDIT‐C) score > 5 categorized as risky drinking.

^d^ Patient Health Questionnaire‐4 score ≥ 3 categorized as psychological distress.

The median BMI in this population was 28.6 kg/m^2^ (IQR: 24.6–32.6). Overall, 27.8%, 32.1% and 40.2% of women had a recorded BMI of < 25.0 kg/m^2^, 25.0–29.9 kg/m^2^ and ≥ 30.0 kg/m^2^, respectively; four had a BMI < 18.5 kg/m^2^ and were therefore classed as underweight. When stratified by ethnicity, women of black African [29.3 kg/m^2^ (IQR: 25.7–33.4)] or other black ethnicity [31.2 kg/m^2^ (IQR: 28.5–34.9)] had higher BMIs than women of white British [24.3 kg/m^2^ (IQR: 21.9–29.4)] or other ethnic backgrounds [24.6 kg/m^2^ (IQR: 22.7–28.2)] (*p* < 0.0001).

Of the 127 women who were categorized as overweight (BMI 25.0–29.9 kg/m^2^), median age was 49 years (IQR: 47–52); prevalence of current smoking and recreational drug use was low (5.5% and 3.1%, respectively); and 56 reported not having enough money to meet their basic needs most or all of the time (44.4%). Almost all (*n* = 121) were on ART.

In univariable analyses, women were more likely to have a BMI ≥ 30.0 kg/m^2^ (and therefore were categorized as obese) if they were of black African [odds ratio (OR) = 2.97, 95% confidence interval (CI): 1.47–6.03] or other black ethnicity (OR = 7.04, 95% CI: 2.47–20.05 vs. white British), were not born in the UK (OR = 1.94, 95% CI: 1.07–3.53 vs. born in the UK) or had a greater number of medical conditions (OR = 1.39, 95% CI: 1.03–1.88 per additional condition present). By contrast, women who reported currently smoking were less likely to have a BMI ≥ 30 kg/m^2^ (OR = 0.37, 95% CI: 0.16–0.87 vs, non‐smokers).

**FIGURE 1:**
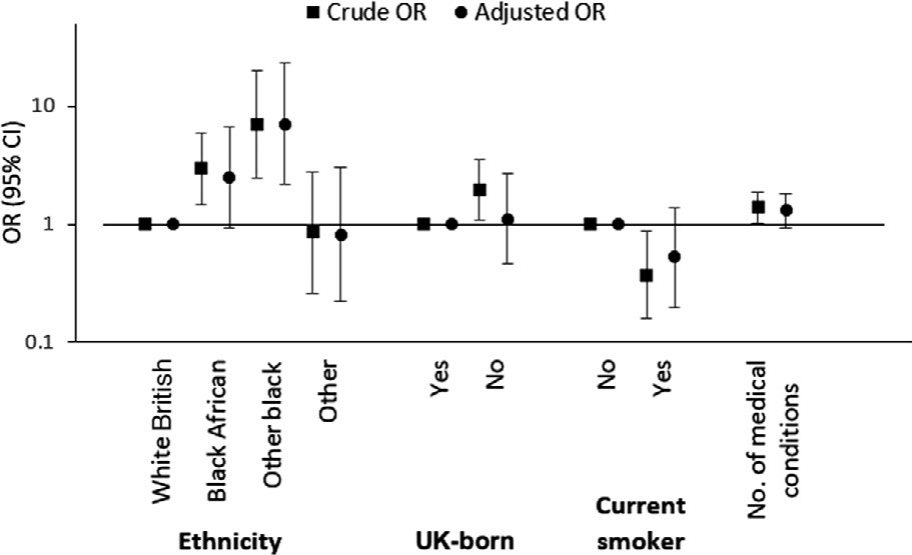
Crude and adjusted odds ratios from logistic regression analyses assessing factors associated with obesity amongst middle aged women living with HIV from the PRIME study. OR, Odds ratio; CI, confidence interval; Number of medical conditions excluding HIV

After adjustment (Figure 1), ethnicity was the only factor that remained significant (black African, OR = 2.51, 95% CI: 0.94–6.71; other black, OR = 7.13, 95% CI: 2.15–23.72; other, OR = 0.82, 95% CI: 0.22–3.06 vs. white British; *p* = 0.002). Associations with country of birth (OR = 1.11, 95% CI: 0.46–2.67 for those born outside the UK vs. those born in the UK; *p* = 0.82), current smoking status (OR = 0.53, 95% CI: 0.20–1.39 vs. non‐smokers; *p* = 0.20) and number of medical conditions (OR = 1.30, 95% CI: 0.93–1.82; *p* = 0.12) were attenuated.

## DISCUSSION

In this analysis of data from 396 women aged 45–60 years attending HIV clinics in England, we found that two‐fifths were obese (BMI ≥** **30 kg/m^2^) and over one‐third were overweight (BMI 25–29 kg/m^2^). These findings are consistent with the 38% prevalence of obesity among 126 women living with HIV in a study conducted in north‐east England, but lower than the 67.8% prevalence reported by Jaff et al. in their study of black South African women aged 40–60 years (of whom 21.3% were women living with HIV) [15, 16].

Of note, we found no evidence of an association between obesity and age, socioeconomic status or behavioural factors. Menopausal status was not associated with obesity in our sample. Only one previous study has explored the association between menopausal status and BMI among women living with HIV, reporting a decrease in BMI among post‐menopausal women [17]. However, that study was conducted in South Africa and only 21.3% of participants were living with HIV; the findings are therefore unlikely to be comparable to those reported in this paper [16, 17]. Finally, unlike recent findings from a UK‐based cohort study of people living with HIV aged ≥ 50 years, we found no association between obesity and CD4 T‐cell count [2].

In our adjusted analysis, women of black African and other black ethnicities were more likely to be obese than women of white British ethnicity, having 2.5‐ and 7‐fold the odds of being obese, respectively. Previous studies in the UK have also reported an association between obesity and black African ethnicity among people living with HIV, whilst in the US Women's Interagency HIV Study (WIHS), African American women were found to have higher BMIs than their white counterparts [2, 15, 18]. The association between elevated BMI and ethnicity is well documented, with 67.5% of black adults in the UK being overweight or obese, a higher proportion than in any other ethnic group [19]. Underlying factors include migration to a more obesogenic environment, cultural factors (including diet, lifestyle factors and physical activity) and socioeconomic status [20]. However, the drivers of obesity among women of black African and other black ethnicities living with HIV have yet to be clearly elucidated.

This analysis is the largest to date in the UK (and one of very few internationally) to explore obesity among middle‐aged women living with HIV, an increasingly important patient group. The sample is broadly representative of women living in the UK and provides insights into BMI in women living with HIV of menopausal age, as well as potential drivers.

However, the PRIME Study is cross‐sectional, meaning we cannot determine directions of some associations and we are unable to comment on trajectory of weight gain over time. Weight and height were missing for 35% of PRIME Study participants, but we did not find any major differences in characteristics between women contributing clinical data to the study who did and did not have available weight and height data. We recognize the limitations of BMI in assessing obesity; anthropometric measurements (e.g. hip and waist circumference) would allow for better assessment of body fat and fat distribution. Menopausal status was categorized by self‐reported menstrual pattern, without biological confirmation, which means that some women may have been misclassified. Our analysis is also limited by lack of detailed data on socioeconomic status (which may explain why we did not find an association between socioeconomic status and obesity in this analysis), and by the absence of data on other potential drivers such as diet, physical activity and food insecurity. Finally, the PRIME study pre‐dates the widespread use of integrase inhibitors; however, we believe that this allows us to explore obesity in this group without the use of these drugs as a confounding factor.

In conclusion, this is the largest study to date in the UK to look at BMI in middle‐aged women living with HIV. We found a high prevalence of elevated BMI, with two in five women being obese. Black African and other black ethnicities were associated with an increased likelihood of being obese, an important finding given that the majority of women living with HIV in the UK are black African. Longitudinal studies are required in order to describe and understand patterns of weight change and direction of associations, as is more detailed exploration of potential mechanisms including physical activity, sleep, diet, socioeconomic status and structural racism. As race and ethnicity are social constructs, black African and other black groups willinclude individuals with considerable diversity in ancestry. Future research should take this into account if looking at genetic mechanisms. Furthermore, qualitative research is needed among middle‐aged women living with HIV to understand perceptions of, and attitudes towards, body shape and to explore potential interventions.

It is likely that obesity and HIV has synergistic effects on the risk of comorbidities such as diabetes, cardiovascular disease and fatty liver disease, all of which are more likely in post‐menopausal women. Our analysis highlights the importance of routine monitoring and recording of BMI in middle‐aged women in clinical practice, in order to identify those with elevated BMI who may benefit from intervention. Moreover, it is important to prioritize work that seeks to develop culturally appropriate and acceptable interventions for ethnically diverse women living with HIV in middle age and beyond, whose needs are likely to differ from those of men living with HIV. This is imperative if we are to address inequalities among people living with HIV in the UK.

## CONFLICT OF INTEREST

CS reports funding from Gilead Sciences, ViiV Healthcare and Janssen‐Cilag for membership of Data Safety and Monitoring Boards, Advisory Boards and for preparation of educational materials. FB has received speaker and consultancy fees from Gilead Sciences. RD has received funding for participation in advisory boards from Gilead Sciences and speaker honoraria from Gilead Sciences, ViiV Healthcare and Janssen‐Cilag. ST has previously received speaker honoraria and funding for preparation of educational materials from Gilead Sciences. AA, HO, LS, KH, RG and FP have no conflicts of interest to declare.

## AUTHOR CONTRIBUTIONS

AA – first author, design of work, initial draft of manuscript. HO – design of work, analysis, interpretation, manuscript writing and corresponding author. KH, LS and RD – design of work, writing and technical editing of manuscript. FB, RG, FP – revision of manuscript. CAS and ST – statistical analysis, writing and technical editing of manuscript, final overseeing of manuscript submission. The authors agreed on all aspects of the work for the final manuscript.
